# Outcome of Femoral Varus Derotational Osteotomy for the Spastic Hip Displacement: Implication for the Indication of Concomitant Pelvic Osteotomy

**DOI:** 10.3390/jcm9010256

**Published:** 2020-01-17

**Authors:** Hoon Park, Sharkawy Wagih Abdel-Baki, Kun-Bo Park, Byoung Kyu Park, Isaac Rhee, Seung-Pyo Hong, Hyun Woo Kim

**Affiliations:** 1 Department of Orthopaedic Surgery, Gangnam Severance Hospital, Yonsei University College of Medicine, 211 Eonju-ro, Gangnam-gu, Seoul 06273, Korea; hoondeng@yuhs.ac (H.P.); weanypooh@yuhs.ac (S.-P.H.); 2 Department of Orthopaedic Surgery, Aswan University Hospital, Aswan University Faculty of Medicine, Aswan 81528, Egypt; sharkawyelbanna@aswu.edu.eg; 3 Division of Pediatric Orthopaedic Surgery, Severance Children’s Hospital, Yonsei University College of Medicine, 50-1 Yonsei-ro, Seodaemun-gu, Seoul 03722, Korea; pedoskbp@yuhs.ac; 4 Department of Orthopaedic Surgery, Inje University Haeundae Paik Hospital, Busan 48108, Korea; yspbk@naver.com; 5 Medical Course, University of Melbourne, Melbourne Medical School, Melbourne 3010, Australia; isaac.rhee@gmail.com

**Keywords:** femoral varus derotational osteotomy, pelvic osteotomy, hip displacement, cerebral palsy

## Abstract

No previous studies have suggested a reliable criterion for determining the addition of a concomitant pelvic osteotomy by using a large patient cohort with quadriplegic cerebral palsy and a homogenous treatment entity of femoral varus derotational osteotomies (VDRO). In this retrospective study, we examined our results of hip reconstructions conducted without a concomitant pericapsular acetabuloplasty in patients with varying degrees of hip displacement. We wished to investigate potential predictors for re-subluxation or re-dislocation after the index operation, and to suggest the indications for a simultaneous pelvic osteotomy. We reviewed the results of 144 VDROs, with or without open reduction, in 72 patients, at a mean follow-up of 7.0 (2.0 to 16.0) years. Various radiographic parameters were measured, and surgical outcomes were assessed based on the final migration percentage (MP) and the Melbourne Cerebral Palsy Hip Classification Scale (MCPHCS) grades. The effects of potential predictive factors on the surgical outcome was assessed by multivariate regression analysis. A receiver operating characteristic (ROC) curve analysis was also performed to determine whether a threshold of each risk factor existed above which the rate of unsatisfactory outcomes was significantly increased. In total, 113 hips (78.5%) showed satisfactory results, classified as MCPHCS grades I, II, and III. Thirty-one hips (21.5%) showed unsatisfactory results, including six hip dislocations. Age at surgery and preoperative acetabular index had no effects on the results. Lower pre- and postoperative MP were found to be the influential predictors of successful outcomes. The inflection point of the ROC curve for unsatisfactory outcomes corresponded to the preoperative MP of 61.8% and the postoperative MP of 5.1%, respectively; these thresholds of the pre- and postoperative MP may serve as a guideline in the indication for a concomitant pelvic osteotomy. Our results also indicate that the severely subluxated or dislocated hip, as well as the hip in which the femoral head is successfully reduced by VDRO but is still contained within the dysplastic acetabulum, may benefit from concomitant pelvic osteotomy.

## 1. Introduction

Hip displacement is one of the most common problems seen in patients with quadriplegic cerebral palsy (CP) [1]. The reported prevalence of hip displacement in children with CP ranges from 1% to 75% [2,3]. Hip subluxation or dislocation is attributed to asymmetrically increased muscle tone and spasticity in conjunction with increased femoral anteversion and progressive valgus deformity of the proximal femur [4]. If untreated, this condition may eventually lead to degenerative arthritis and induce intractable pain [4,5,6]. The primary goals of treatment for the spastic hip diseases are obtaining painless, flexible, and well-located hips to facilitate easy transfer of the patients, and their comfort sitting in a wheelchair [3]. Hip surveillance programs for children with CP have been adopted to prevent dislocation by early detection and early preventive surgery [7]. In both the Australian and Swedish settings, the prevalence of late dislocation had decreased, and the need for salvage surgery has been reduced [8,9]. Reconstructive surgeries to correct myostatic muscle deformities and femoral/acetabular malalignments are recommended when there is no severely deformed femoral head caused by a chronically dislocated hip [10].

Osteotomy options include femoral varus derotational osteotomy (VDRO) and a combination of both VDRO with pericapsular acetabuloplasty [1,3,4]. Some have reported favorable outcomes in patients who underwent isolated VDRO without open reduction [11,12,13]. Additional pelvic osteotomy to avoid revision was not observed to be necessary in all patients [3,10], and it produced frequent complications and required longer recovery times [14]. Another concern for the necessity of the acetabular procedure was based on the changes in the mechanics caused by VDRO which can subsequently lead to spontaneous acetabular remodeling over time [1,15,16]. On the other hand, studies comparing the results of isolated VDRO with those of combined procedures suggest that pelvic osteotomy should be considered in patients with higher preoperative migration percentage (MP) [17,18,19,20,21]. However, they obtained those values based only on the lowest preoperative MP measured in their patients who had had re-subluxations or dislocations; they explained the effectiveness of the procedure by simply reporting the results from a subset of patients enrolled in their study.

The debate on whether to perform an isolated VDRO or to combine a pelvic osteotomy is still discussed [11,16], and the indications for a simultaneous pelvic osteotomy in a condition as complex as spastic hip displacement are still unclear. A review of the literature is difficult because there is a wide spectrum of neurologic involvement and ambulatory statuses in the literature. Furthermore, a wide variety of procedures are used and the degrees of hip displacement and acetabular dysplasia at the time of surgery differ among the studies. To date, none of the studies suggest a reliable criterion for determining the need of a concomitant pelvic osteotomy by using a large patient cohort with similar ambulatory mobility and a homogeneous treatment entity, such as hip reconstruction without pelvic osteotomy.

In this retrospective study, we examined our results of hip reconstructions conducted without a concomitant pericapsular acetabuloplasty in patients with varying degrees of hip displacement. The primary aim of the study was to identify any prognostic factors affecting the outcomes. The secondary aim was to predict the probability of re-subluxation or re-dislocation after the index operation, and to suggest the indications for a simultaneous pelvic osteotomy. We hypothesized that hip reconstructions without a concomitant pelvic osteotomy would not achieve a successful outcome in all patients, and that a threshold of any potential clinical and radiologic factors would predict surgical failure.

## 2. Experimental Section

### 2.1. Subjects

This retrospective study was approved by our Institutional Review Board (IRB No. 1-2015-0016). The IRB of our hospital waived the need of receiving informed consent from the patients; our research involved no more than a minimal risk to our subjects and we used the existing medical records and the radiographs. Between September 2000 and December 2015, the senior surgeon performed various types of hip reconstructive surgery in 338 patients with spastic CP with hip displacements. The surgical indication was a subluxated or dislocated hip and the resultant difficulties were with sitting balance and perineal care. The inclusion criteria for the present study were as follows; (1) patients classified as having level IV or V of mobility according to the Gross Motor Function Classification System (GMFCS) [22], (2) hips with MP > 30% treated by bilateral VDROs without concomitant pelvic osteotomy, and (3) availability of plain radiographs of the hip and spine for the follow-up review. Patients were excluded when (1) previous surgeries had been done elsewhere on the femur and/or the pelvis before the index operation, (2) the duration of follow-up was less than 2 years, and (3) there was a concomitant severe scoliosis and pelvic obliquity required a spinal fusion as it may act as a potential confounder for the analysis [23].

We first excluded 246 patients whose simultaneous pelvic osteotomy was done at least on one side of the hips at the time of VDRO, and 10 patients were also excluded due to inadequate clinical and radiological follow-up. Ten patients were further excluded as they had severe scoliosis and pelvic obliquity that required spinal fusions. A total of 144 displaced hips in 72 patients (48 males and 24 females) were included in the current study. All patients underwent bilateral operations in one surgical setting. VDRO with or without open reduction had been the choice of treatment in our institution until 2008 and the majority of our enrolled patients were cases before this time. As such, little selection bias was present and our results represented the outcome of the index operations performed in hips with varying degrees of displacement. The GMFCS level was IV in 40 patients and level V in 32. The mean age at the time of surgery was 6.2 (3.2 to 12.2) years, and the average follow-up period was 7.0 (2.0 to 16.0) years.

### 2.2. Surgical Procedures

All surgical procedures were performed in one surgical setting. For dislocated hips, a traditional open reduction through an anterior approach to the hip, followed by VDRO, was carried out. For subluxated hips, open tenotomies of the adductor longus and gracilis and anterior neurectomy of the obturator nerve were firstly completed through a small transversal incision made in the groin. VDRO was then performed through a lateral approach to the proximal femur. An angled blade plate (DePuy Synthes, West Chester, PA, USA) was used to fix the osteotomy. The usual neck-shaft angle after fixation with a plate was about 100° to 110° and the anteversion was 10° to 15°. Any degrees of varus angulation and anteversion were adjusted to try to achieve a concentric reduction of the femoral head within the acetabulum. An extension component at the osteotomy was also added if necessary.

After placing a chisel in the femoral neck and making an osteotomy at the intertrochanteric level, the psoas tendon was completely released and the lesser trochanteric apophysis was excised en bloc. After temporary fixation of the osteotomy, the quality of the reduction was checked with the aid of an intraoperative fluoroscopic arthrogram and by confirmation of restoration of the Shenton line. When the femoral head was not found to be concentrically located within the joint, we performed a medial capsulotomy of the hip joint after creating an interval between the glutei and tensor fascia lata. We frequently encountered the need to adjust degrees of varization/derotation or the need to add a few millimeters of femoral shortening in these situations. If there were residual limitations of abduction after the reduction of a femoral head and fixation with a plate, an additional release of the adductor brevis and/or the medial hamstrings were performed, as necessary. This was so that hip abduction of 35–40 degrees could be achieved. For the 43 hips in which the first trial of VDRO and soft-tissue releases failed to pull the femoral head sufficiently down to the level of the acetabulum, we proceeded directly with an open reduction through the anterior approach, as done in dislocated hips.

The patients were immobilized in a hip spica cast after surgery for a total of 4–6 weeks. After removal of a cast, comprehensive rehabilitation program was initiated and a hip abduction brace with the hip extended was worn during napping hours and night-time.

### 2.3. Outcome Assessment

Anteroposterior pelvic radiographs, taken with the hips slightly internally rotated, were used for radiological measurements. All measurements were performed by two orthopedic surgeons (B.K.P. and S.-P.H.). Preoperative, postoperative, and final follow-up radiographs were used and the migration percentage (MP) [24], acetabular index (AI) [25], femoral neck-shaft angle (NSA), and head-shaft angle (HSA) [26] were measured. Hip subluxation requiring reconstructive surgery was defined according to the current literature as MP > 30% [1,2,10,11,16,27,28,29,30], and dislocation was defined as MP of 100%. AI was measured in 94 hips in 47 patients, and it could not be measured in the remaining 25 patients, as the triradiate cartilage had already been fused at the time of final follow-up. The geometry of the proximal femur was assessed by NSA as well HSA. HSA is less affected by the degrees of hip rotation [31], and we used this angle to monitor subsequent remodeling of the proximal femur during the follow-up period. The presence and severity of avascular necrosis (AVN) was evaluated according to the classification system by Kruczynski et al. [32]. The degrees of pelvic obliquity, the angle made by the horizontal line and the line between the lowest points of the pelvic bones on the right and left sides, was also checked.

**Figure 1:**
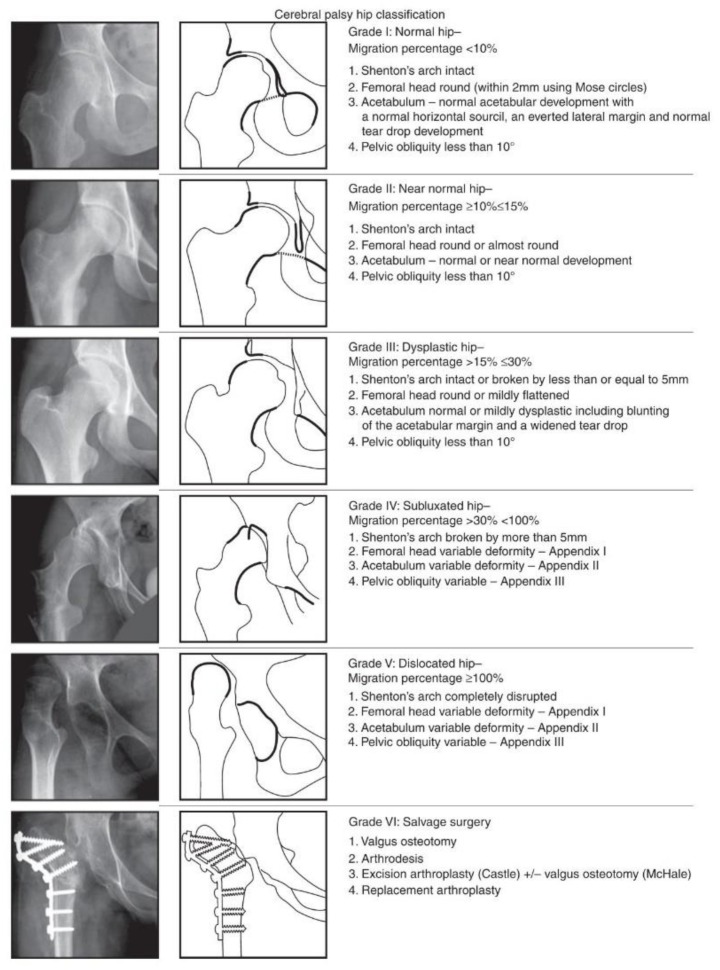
Radiographs and diagrams showing The Melbourne Cerebral Palsy Hip Classification Scale (reproduced) [30].

The Melbourne Cerebral Palsy Hip Classification Scale (MCPHCS) [30] was used to evaluate the overall developmental status of the hip joint, before surgery and at the final follow-up (Figure 1). The MCPHCS is a categorical, radiographic classification of hip morphology based on qualitative indices (the integrity of the Shenton line, shape of the femoral head, shape of the acetabulum, and pelvic obliquity) and measurement of a key continuous variable (the migration percentage of Reimers); it is a valid and a reliable tool to examine hip morphology in CP [33,34].

### 2.4. Statistical Analysis

Statistical analyses were performed using SPSS^®^ version 25.0 (SPSS, Chicago, IL, USA), with significance defined as *p* < 0.05. Inter-rater and intra-rater reliability of each radiologic parameter was evaluated using an intraclass correlation coefficient (ICC); an ICC between 0 and 0.20 was considered to be low, 0.21 to 0.40 fair, 0.41 to 0.60 moderate, 0.61 to 0.8 good, and 0.81 to 1 to be excellent concordance. Data were assessed for normality on plots and with the Shapiro—Wilk test. Repeated measures analysis of variance was utilized to examine the changes in radiologic parameters over three time points; preoperative, immediate postoperative, and final follow-up. Paired-*t* test was used to compare preoperative and postoperative variables.

In the risk analyses, clinical and radiographic variables were examined; age at the time of surgery, sex, preoperative MP, preoperative AI, postoperative MP, postoperative NSA and HSA were included as potential risk factors that would be related to the final outcome. These were chosen based on the findings of previous studies suggesting potential factors related to the hip joint status after any reconstructive hip surgeries in CP [10,11,14,16,17,18,19,27,35], and were in line with our hypotheses. To examine the effects of these variables on the MCPHCS grades at the final follow-up, we used a univariate and multivariate general linear mixed model. This model was chosen due to the potential influences of bilaterality. Additionally, we evaluated the influences of the above-mentioned risk factors on a satisfactory outcome which was defined as MCPHCS grades of I, II, and III. For this, univariate and multivariate generalized estimating equation models were constructed by considering the paired hip data. In each univariate model, all potential risk factors were analyzed, and variables identified as significant in the univariate analysis, with a *p* value < 0.05, as well as clinical variables were included in the multivariate analysis.

A receiver operating characteristic (ROC) curve was used to determine cut-off values for each risk factor at the final follow-up that distinguished between the hips with a satisfactory outcome and those with an unsatisfactory outcome. The inflection point on this curve represents the value with the highest sensitivity and specificity and was chosen as the threshold of each risk factor. Statistical significance of the ROC curve was determined using an area-under-the-curve (AUC) analysis, with a probability of less than 0.05 considered to be statistically significant.

## 3. Results

Before surgery, hip subluxations were noted in 89 hips of 60 patients and dislocations in 38 hips of 26 patients. Bilateral dislocations were present in 12 patients. At the time of final follow-up, re-subluxations or dislocations of the hip were found in 31 (21.5%) hips in 26 patients. Six re-dislocations occurred in all cohorts; two painful hips were treated with subtrochanteric valgus osteotomy with or without femoral head resection, and the remaining four hips were left untreated, as the parents refused re-operation. Radiological signs of AVN were observed in 38 (26.3%) hips: 17 hips with grade I; 13 grade II; two grade III, four grade IV, and two grade V. Four hips classified as having grade IV of AVN were re-subluxated and two hips classified as grade V were dislocated eventually. Minor complications were encountered, including seven superficial cast ulcers and six superficial wound infections which resolved without additional major surgical intervention.

The MP, AI, NSA, and HSA showed good to excellent intra-observer and inter-observer reliabilities (Table 1). These results were comparable to the values reported in the literature [36,37,38].

Overall radiologic results, including hips classified by MCPHCS grades, are summarized in Table 2. The average preoperative MP was 62.0% and it improved to 12.8% postoperatively (*p* < 0.001). At the final follow-up, MP significantly increased to 27.9% (*p* < 0.001). The mean AI was 22.8° before surgery and was significantly decreased to 19.3°, where 94 hips were available to measure at the final follow-up (*p* < 0.001). The average NSA and HSA significantly decreased after surgery, but were examined to be increased at the final follow-up (*p* < 0.001, respectively), indicating remodeling of the proximal femur into valgus position again over time. One hundred thirteen hips (78.5%) in 67 patients had a satisfactory outcome, classified as MCPHCS grades I, II, and III.

In the multivariate linear mixed model, the pre-and postoperative MP were found to be closely related to the improvement in the MCPHCS grades (*p* = 0.04 and *p* = 0.002, respectively). Age at surgery, sex, preoperative AI, postoperative NSA, and postoperative HSA were not associated with the improvement in the MCPHCS grades (Table 3).

A multivariate generalized estimating equation revealed the lower preoperative MP (OR = 0.98, 95% CI = 0.95 to 0.99, *p* = 0.03) and the lower postoperative MP (OR = 0.96, 95% CI = 0.94 to 0.99, *p* = 0.007) were significant predictors for a satisfactory outcome. The odds ratios for preoperative and postoperative MP per 10 units were 0.78 and 0.71, respectively. Age at surgery, sex, preoperative AI, postoperative NSA, and postoperative HSA were not observed to be the predictors of a satisfactory outcome (Table 4).

**Figure 2:**
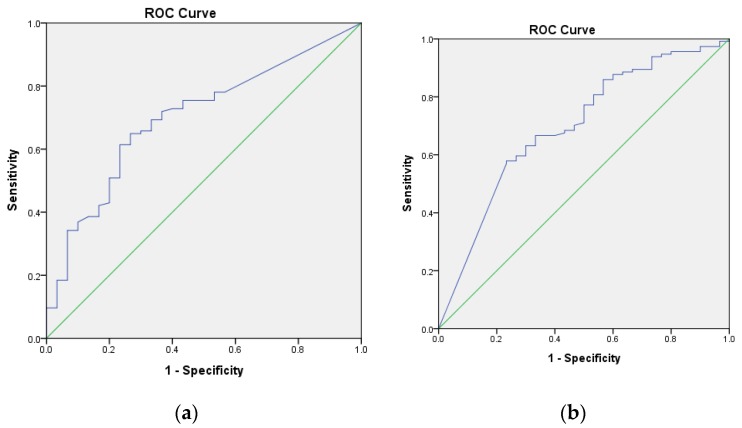
(**a**) The receiver operating characteristic (ROC) curve was used to determine a threshold level of the preoperative migration percentage (MP) above which the risk of unsatisfactory surgical result was significantly elevated. The inflection point of the curve corresponded to the preoperative MP of 61.8%; this represents the value with the highest sensitivity and specificity and was thus chosen as the preoperative MP. The area under the curve (AUC) is represented by the area of the graph beneath the blue line. (**b**) The ROC curve was used to determine a threshold level of the postoperative MP above which the risk of unsatisfactory surgical result was significantly elevated. The inflection point of the curve corresponded to the preoperative MP of 5.1%. The AUC is represented by the area of the graph beneath the blue line.

ROC curves were used to determine cut-off values of pre-and postoperative MP. The preoperative MP of 61.8% or lower was noted to be the threshold for having a satisfactory outcome, with 65% sensitivity and 74% specificity (AUC = 0.700, 95% CI = 0.601 to 0.79, *p* = 0.001) (Figure 2a). Postoperative MP of 5.1% or lower was noted to be the threshold for having a satisfactory outcome, with 58% sensitivity and 77% specificity (AUC = 0.696, 95% CI = 0.589 to 0.803, *p* = 0.001) (Figure 2b).

## 4. Discussion

The ideal surgical treatment of hip displacement in quadriplegic CP is to obtain concentric reduction and to maintain a stable reduction against the life-long spasticity present in the patient. Adequate soft-tissue releases and VDRO have been regarded as the primary treatments in most spastic hip displacement. However, the necessity of additional procedures, such as open reduction at the time of VDRO and of simultaneous pelvic osteotomy, has not been scrutinized in a systemic manner. In our series of VDRO, the senior surgeon had applied a sequential approach according to the severity of hip displacement present before surgery and the degrees of containment of the femoral head after temporary fixation of an osteotomy, as determined by intraoperative arthrogram. By adapting this approach, we could determine the longevity of the successfully carried-out VDRO, with or without open reduction, for the hips with varying degrees of displacement and acetabular dysplasia.

In order to suggest a criterion for a concomitant acetabuloplasty, we first assessed the overall results of the index operation; the re-subluxation rate based on the MCPHCS was 21.5%. The reported rate of re-subluxation after isolated VDRO (without open reduction) ranged from 10% to 74% [11,12,13,19,39,40]. Series of combined procedures on the femoral and the acetabular sides showed the rate of re-subluxation to be from 2% to 41% [14,27,41,42,43]. It is difficult to compare our results directly with others due to the heterogeneity based on the degrees of preoperative hip displacement, the mobility of patients, the types of surgery, the criteria for poor results, and the duration of follow-up. Nevertheless, even if the 38 (26.4%) completely dislocated hips were included in the present study, our results were comparable to those of previous observations on the procedure performed in mostly subluxated hips.

We found that both the preoperative MP of 61.8% and the postoperative MP of 5.1% were the thresholds above which the risk of surgical failure increases significantly. Therefore, the subset of patients above these thresholds may be indicated for a simultaneous pelvic osteotomy for correction of a residual acetabular deficiency. With regards to preoperative MP, there were studies suggesting its values ranged from 50% to 80% in terms of the necessity of simultaneous acetabuloplasty [18,19,35]. However, all of the studies estimated those values solely based on the lowest preoperative MP in patients with re-subluxations or dislocations, and they did not conduct a formal statistical analysis. In the current study, we determined the threshold values with ROC analysis and we believed that our criterion is more reliable than those of previous suggested.

Traditionally, preoperative MP has been regarded as the only determinant in choosing the most suitable reconstructive hip surgery [11,18,19,27]. As experienced by others, we observed that a larger preoperative hip displacement resulted in a more unsatisfactory outcome of the index operation. This may be because it is more technically difficult to obtain concentric reduction in patients with larger hip displacement. However, we believe that careful consideration of the degrees of preoperative hip displacement, as well as the intraoperative status of a reduced hip, is of importance in determining the addition of a concomitant pelvic osteotomy. In the congenitally dysplastic hips treated with varizational osteotomy performed at four years of age or younger, the presence of a concentrically reduced femoral head was a prerequisite for anticipating the maintenance of a reduced hip and subsequent acetabular remodeling [44]. On the other hand, the above findings may not be true of severely subluxated or dislocated hips in CP; the widened teardrop area and the condyloid cavity of the acetabulum filled medially with hypertrophic cartilage may force the femoral head laterally over time. Our observation of a lower postoperative MP < 5.1%, as another predictor of the satisfactory outcome, indicates that an additional acetabular procedure should be considered even in the well-reduced hips but with suboptimal coverage of the femoral head by the deficient acetabulum.

Taking into account that the MP may increase with age [45], early intervention with a VDRO may prevent the need for a more invasive surgery [13,16,46]. However, there is an inconsistency as to the influence of age on the results of various types of surgery [19,27,35,38]. Furthermore, there is still controversy around optimal timing of an operative intervention for the hip reconstruction. Surgery at a younger age can lead to better results because younger children have greater remodeling potential of the acetabulum [13,16,46]. On the contrary, delaying surgery at an older age has been supported by others [7,38,39] due to rebound coxa valga seen in patients treated with VDRO. Although age at the time of our index operation was found to be not a predictive factor for the satisfactory outcome, we think that the discrepancy on the age effects among the studies may be due to multiple factors; such as altered potential of the acetabular remodeling in patients with CP and progressive valgus deformity of the proximal femur caused by persistent weakness of the hip abductors resulting in growth retardation through the greater trochanter [47].

In the present study, we observed a significant improvement in average AI at the final follow-up. However, the change was only 3.5 degrees and we did not find a correlation between preoperative AI and surgical success. Some authors have advocated the preoperative AI as an indicator for performing acetabuloplasty [14,17,46]. However, AI may not be the best radiographic parameter for the assessment of acetabular integrity in CP, as it varies with pelvic orientation and rotational malposition, decreases with lumbar lordosis, and increases with hip flexion [48]. Most acetabular deficiencies are posterolateral or global in spastic hip displacement and their exact location is less identifiable on supine radiographs; thereby making it difficult to accurately measure the AI [49]. Furthermore, the triradiate cartilage in many of our hips had been fused at the time of the last follow-up, hence we could not measure AI.

Contrary to the past studies, we assessed our surgical outcomes with the MCPHCS. Although MP has been the most commonly used radiologic measurement to describe hip displacement in CP, MCPHCS provides more comprehensive information about the hip morphology in addition to the MP; including integrity of the Shenton line, roundness of the femoral head, development of the acetabulum, and degrees of the pelvic obliquity [30]. The inter-and intra-observer reliability of the system have been demonstrated to be excellent to almost perfect [33,50].

There are several limitations to this study. First, although we suggested the pre-and postoperative MP values as an indication for a pelvic osteotomy, we are not aware if an additional pelvic osteotomy in those patients meeting the criteria we have provided may have always satisfactory results. However, under appropriate indications, we can assume better surgical outcomes with the use of a concomitant pelvic osteotomy. Second, as we wished to investigate the long-term effects of VDRO only without concomitant pelvic osteotomy, we excluded the patients who were treated with simultaneous pelvic osteotomy on at least one side of the hip; hence, there is a possibility that this selection bias might have affected our study. Third, limitations of a retrospective study design allowed for varying follow-up times between the patients. The differing follow-up times may affect the outcomes and rates of complication. Additionally, as some patients were relatively young at the final follow-up and had not yet reached skeletal maturity, future re-migration or re-dislocation could be possible and further follow-up is needed to determine long-term results. And finally, while the main indications for surgery were the prevention of pain and improved femoral head reduction within the acetabulum, no functional hip score was measured due to the absence of a validated functional instrument available for the study on the spastic hip. However, Wawrzuta et al. found that the pain’s severity and frequency was related to the hip morphology, stating that patients with excellent or good hip development, classified as having Grade I-III, had less pain compared to those with fair (Grade IV) and poor (Grade V-VII) hip development [34]. We believe that improved radiographic parameters after index operation may predict decreased hip pain or improved function in this patient population.

## 5. Conclusions

A preoperative MP > 61.8% and the postoperative MP > 5.1% were inflection points for hip re-subluxation and re-dislocation after hip reconstruction without pelvic osteotomy in non-ambulatory CP patients. These findings indicate that severely subluxated or dislocated hips, as well as hips in which the femoral head is successfully reduced by VDRO but is still contained within the dysplastic acetabulum, may benefit from additional pelvic osteotomy. Threshold values for the pre- and postoperative MP can serve as a guideline in the selection of the concomitant pelvic osteotomy.

## Figures and Tables

**Table 1:** Inter-rater and intra-rater reliabilities of the radiographic measurements.

Measurement	Inter-Rater Reliability		Intra-Rater Reliability	
	ICC	95% CI	ICC	95% CI
Migration percentage	0.97	0.96–0.99	0.92	0.90–0.94
Acetabular index	0.81	0.76–0.86	0.78	0.70–0.82
Neck-shaft angle	0.94	0.91–0.96	0.91	0.87–0.93
Head-shaft angle	0.88	0.83–0.93	0.84	0.81–0.90

ICC, intraclass correlation coefficients; CI, confidence interval.

**Table 2:** Radiological measurements.

Variables	Preoperative	Postoperative	Final Follow-Up
Migration percentage (%)	62.0 ± 27.9	12.8 ± 15.5	27.9 ± 19.6
Acetabular index (°)	22.8 ± 4.1	N/A	19.3 ± 4.3
Neck-shaft angle (°)	154.4 ± 9.9	125.7 ± 10.6	138.5 ± 17.2
Head-shaft angle (°)	164.5 ± 10.1	133.9 ± 11.3	147.4 ± 13.7
MCPHCS grade (no. (%))			
I	0		19 (13.2%)
II	0		31 (21.5%)
III	0		63 (43.8%)
IV	106 (73.6%)		25 (17.3%)
V	38 (26.4%)		4 (2.8%)
VI	0		2 (1.4%)

Values of percentage, index, and angle are expressed as the mean ± SD.

**Table 3:** Factors affecting the final Melbourne Cerebral Palsy Hip Classification Scale (MCPHCS) grade using linear mixed model.

Factors	Univariate		Multivariate	
	Coefficient ^1^	*p*-Value	Coefficient ^1^	*p*-Value
Age at surgery	−0.002 (−0.1 to 0.1)	0.9	0.04 (–0.08 to 0.15)	0.5
Sex	0.01 (−0.4 to 0.4)	0.9	−0.15 (−0.6 to 0.3)	0.5
Preoperative MP	0.01 (0.005 to 0.02)	0.001	0.008 (0 to 0.02)	0.04
Preoperative AI	0.07 (0.02 to 0.12)	0.004	0.04 (−0.01 to 0.09)	0.14
Postoperative NSA	−0.02 (−0.04 to 0.01)	0.2		
Postoperative HSA	0.01 (−0.01 to 0.04)	0.2		
Postoperative MP	0.03 (0.01 to 0.04)	<0.001	0.02 (0.008 to 0.03)	0.002

^1^ Values are given as coefficient, with the 95% CI enclosed in parentheses. MCPHCS, Melbourne Cerebral Palsy Hip Classification Scale; MP, migration percentage; AI, acetabular index; NSA, neck shaft angle; HSA, head shaft angle; CI, confidence interval.

**Table 4:** Predictors of surgical success using generalized estimating equation.

Factor	Univariate		Multivariate		Multivariate	
	Odds Ratio ^1^	*p*-Value	Odds Ratio ^1^	*p*-Value	Odds Ratio ^1^	*p*-Value
Age at surgery	0.9 (0.8 to 1.2)	0.9	0.9 (0.7 to 1.1)	0.3	0.9 (0.7 to 1.1)	0.3
Sex	1.1 (0.5 to 2.4)	0.9	1.7 (0.6 to 4.7)	0.3	1.7 (0.6 to 4.7)	0.3
Preoperative MP	0.98 (0.96 to 0.99)	0.005	0.98 (0.95 to 0.99)	0.03		
Preoperative MP (per 10 unit)	0.78 0.65 to 0.93)	0.005			0.78 (0.61 to 0.98)	0.03
Preoperative AI	0.87 (0.78 to 0.97)	0.013	0.9 (0.8 to 1.0)	0.07	0.9 (0.8 to 1.0)	0.07
Postoperative NSA	1.03 (0.97 to 1.1)	0.36				
Postoperative HSA	0.99 (0.95 to 1.03)	0.68				
Postoperative MP	0.96 (0.94 to 0.98)	0.001	0.96 (0.94 to 0.99)	0.007		
Postoperative MP (per 10 unit)	0.67 (0.53 to 0.86)	0.001			0.71 (0.55 to 0.91)	0.007

^1^ Values are given as coefficient, with the 95% CI enclosed in parentheses. MP, migration percentage; AI, acetabular index; NSA, neck shaft angle; HSA, head shaft angle; CI, confidence interval.
